# Automated Classification of Dental Caries in Bitewing Radiographs Using Machine Learning and the ICCMS Framework

**DOI:** 10.1155/ijod/6644310

**Published:** 2025-08-21

**Authors:** Mehdi Salehizeinabadi, Saghar Neghab, Nazila Ameli, Kasra Koucheh Baghi, Camila Pacheco-Pereira

**Affiliations:** ^1^Department of Dentistry, Mike Petryk School of Dentistry, University of Alberta, Edmonton, Alberta, Canada; ^2^Department of Dentistry, School of Dentistry, University of Toronto, Toronto, Ontario, Canada; ^3^Private Practice, Toronto, Ontario, Canada

**Keywords:** artificial intelligence, bitewing radiographs, dental caries, machine learning, YOLO network

## Abstract

**Background:** Dental caries is considered a public health issue, with early detection being crucial for effective management. Traditional diagnostic methods, including visual examination and bitewing radiographs, are prone to interpretation variability. Artificial intelligence (AI), particularly deep learning (DL), has shown promise in improving diagnostic accuracy. This study evaluates the YOLOv11 model for dental caries detection and segmentation in bitewing radiographs, using the standardized International Caries Classification and Management System (ICCMS) framework.

**Methods:** A dataset of 730 bitewing radiographs, containing 1115 annotated carious lesions, was used for training and validation. Annotation was performed by experienced dentists using the Roboflow platform. To evaluate annotation consistency, a subset of 10 images was independently annotated by both dentists. Agreement was assessed using Intersection over Union (IoU) and Dice similarity coefficient (DSC). The YOLOv11 model was trained for 50 epochs with data augmentation techniques. Performance was assessed using precision (P), recall (R), and mean average precision at 50% IoU (mAP50).

**Results:** The reliability analysis showed strong agreement, with an average interrater IoU of 0.82 and DSC of 0.85, and intrarater IoU of 0.84 and DSC of 0.87 across the 10 images. The YOLOv11 model excelled in detecting and segmenting advanced carious lesions, achieving high mAP50 values of 0.74 and 0.80 for RB4 + RC5 and RC6 classes, respectively. However, it showed moderate performance for early-stage lesions (RA1 + RA2 and RA3), with mAP50 scores of 0.61 and 0.52, respectively. This disparity highlights areas for potential enhancement through additional data augmentation and model fine-tuning.

**Conclusion:** The YOLOv11 model is highly effective in identifying dental caries, especially advanced lesions, but struggles with detecting early stages of caries. AI enhancements could improve diagnostic accuracy, enable better early interventions and improve patient outcomes. The research supports incorporating AI technologies into dental radiographic evaluations to improve diagnostics and clinical results.

## 1. Introduction

Dental caries, or tooth decay, constitutes a significant public health issue affecting millions globally, transcending age, socioeconomic status, and geographic boundaries [1, 2]. As a pandemic of sorts, dental caries contributes to significant public and private healthcare expenditures [3]. It is primarily a multifactorial disease influenced by dietary habits, fluoride intake, and the presence of bacterial biofilms that promote demineralization and lesion progression [4–6].

Conventionally, dental caries detection has employed methods such as visual and tactile examinations coupled with bitewing radiographs [7, 8]. This traditional method, while foundational, is fraught with limitations including the potential for misinterpretation as dentists have historically had trouble with accurately detecting radiographs for proximal dentin caries more so than for occlusal caries [9, 10]. Such challenges underscore the necessity for more nuanced diagnostic approaches that can detect early signs of demineralization, particularly in populations at elevated risk [11].

To implement and secure standardization, the International Caries Classification and Management System (ICCMS) has a standardized framework for the diagnosis, assessment, and management of carious lesions [12]. It incorporates a radiographic scoring system (RSS) that categorizes the extent of carious penetration in posterior teeth, facilitating more informed treatment decisions aimed at mitigating caries susceptibility [13].

Amidst these developments, the role of artificial intelligence (AI) in enhancing dental diagnostic procedures has been increasingly recognized [14–17]. Deep learning (DL) techniques are being widely applied for quick evaluation of dental radiographs, without subjective interpretations. The intelligent systems can perform these tasks through the process of medical image understanding [18, 19]. AI technologies, particularly those involving DL models like You Only Look Once (YOLO), have demonstrated exceptional capabilities in object detection tasks across various fields, including medical imaging [12]. The YOLO framework operates by segmenting image pixels into discrete bounding boxes and evaluating the probability of class presence, thereby allowing for precise localization of objects [20] in this case, carious lesions. The latest iteration, YOLOv11, represents a significant advancement in object detection technology, introducing architectural improvements designed to enhance detection speed, accuracy, and robustness in complex environments [21].

Preliminary studies utilizing earlier versions of YOLO in dentistry have reported high accuracy rates in tasks such as detecting dental anomalies in various types of radiographs [22]. These findings suggest that its methodologies could surpass traditional diagnostic techniques in identifying carious lesions with greater reliability and less time [23]. The integration of ng YOLO-v11's advanced segmentation capabilities with the rigorous standards set forth by ICCMS could significantly enhance the diagnostic P of dental caries. In their 2023 study, Panyarak et al. [24] explored advancements in caries detection using YOLOv7 in bitewing radiographs, significantly outperforming earlier YOLO models in P for advanced lesions. However, they encountered limitations in detecting early-stage caries, particularly initial enamel lesions. Our study addresses this gap by focusing on enhancing the model's sensitivity to early-stage caries and improving overall classification accuracy. This refinement aims to equip clinicians with more accurate tools for early diagnosis and intervention in dental care. However, previous studies missed using the appropriate radiographic technique for interproximal caries—the bitewing radiograph. Therefore, this research project aims to explore the application of YOLO-v11 in the segmentation and classification of dental caries, as delineated by the ICCMS standards.

## 2. Materials and Methods

This study was approved by the Health Research Ethics Board at the University of Alberta (Pro00144856). To comply with the ALARA principle and follow the Declaration of Helsinki, no radiographs were taken for this study. From an initial pool of 1012 bitewing radiographs, 730 were selected based on quality assessments, excluding those with poor resolution or large restorative fillings and crowns that could interfere with caries detection.

### 2.1. Annotation Process

Annotation was performed using the Roboflow platform [25], where carious lesions were manually outlined on the radiographs using a polygon tool. The initial annotations were performed by a dentist with over 5 years of clinical experience. These were then reviewed and revised by a senior dentist with more than 10 years of clinical practice. The senior dentist's role involved editing and resolving any discrepancies to ensure high-quality and reliable annotations.

To evaluate the agreement between annotators, a subset of 10 randomly selected bitewing radiographs was independently annotated by calibrated dentists. To assess the consistency of the main annotator over time, an intrarater reliability analysis was also conducted by reannotating the same 10 images after a washout period of 1 week. Inter- and intrarater reliability were quantified using the Intersection over Union (IoU) for the segmented carious lesions. Additionally, the DSC, another standard metric for spatial overlap, was calculated to secure reliability of the annotation process.

Following the annotation process, the evaluators proceed with the main sample. For these the lesions were categorized into four classes: RA1 and RA2 were grouped as one class, while RA4 and RB5 were combined into another, resulting in a total of four distinct segmentation classes.

### 2.2. Preprocessing and Augmentation

To enhance variability and improve model generalization, image augmentation techniques were applied to the dataset. The images were cropped to remove unnecessary background and standardized for consistency. The dataset was split into training (60%), validation (20%), and test (20%) sets, prior to any augmentation. A total of 147 images were kept completely independent as test data and were never subjected to any augmentation or exposure during model training. Data augmentation was performed exclusively on the training and validation sets after the initial split to ensure that no augmented image derived from a test image was included in the training process. This approach effectively eliminated any risk of data leakage and ensured a fully independent test set for reliable model evaluation. Augmentation included variations in brightness, horizontal flipping, and rotation at different angles. These preprocessing steps increased the total number of bitewing radiographs to 2190 images, including 3345 caries (with RA1 and RA2: 1107, RA3: 855, RB4 and RC5: 816, and RC6: 567).

### 2.3. Model Architecture and Training

For this study, a segmentation model based on the YOLO-v11 architecture was utilized. This model is optimized for detecting and segmenting objects efficiently, making it well-suited for identifying dental caries in radiographic images. The model consists of 355 layers and over 10 million parameters, designed to balance accuracy and computational efficiency. It processes images through several stages. First, it extracts features using multiple convolutional layers that detect patterns such as edges and textures. It then uses C3k2 blocks, which are specialized layers that help in identifying complex structures in the image while keeping the model lightweight. The feature extraction process involves progressively increasing the number of channels from 32 to 512, allowing the model to capture detailed image information.

To improve performance, the model includes a spatial pyramid pooling-fast (SPFF) module, which helps detect small details by combining information from different parts of the image. Additionally, a cross-stage partial attention (C2PSA) module is used, which applies an attention mechanism to focus on important areas, improving segmentation accuracy [26].

For segmentation tasks, the model generates segmentation masks alongside bounding boxes to highlight areas of interest. These masks are downsampled by a factor of four to reduce memory usage while maintaining P. The model was trained for 50 epochs with an input image size of 320 × 320 pixels. It used the AdamW optimizer with an initial learning rate of 0.00125 and momentum of 0.9 to fine-tune its weights. The training process incorporated multiple loss functions, including IoU-based loss for object localization, Dice loss for segmentation, and focal loss to ensure accurate classification.

To improve generalization, various data augmentations were applied, such as random flipping, brightness adjustments, and Gaussian blur. The model was trained using automatic mixed precision (AMP), which speeds up training by optimizing memory usage on the GPU. Additionally, for optimization, YOLOv11 utilizes the Stochastic gradient descent (SGD) optimizer with momentum, which helps accelerate convergence and navigate the loss landscape more effectively. After training, the model was validated on 147 test images containing 291 instances of caries.

### 2.4. Performance Evaluation

To evaluate the performance of the YOLO11s-seg model, we conducted a thorough assessment using standard object detection and segmentation metrics. The evaluation process involved measuring the model's ability to accurately detect and segment dental caries in radiographic images. Key metrics included P, recall (R), and mean average precision at 50% IoU (mAP50).

The model was validated on a separate dataset including 147 new images, ensuring that performance was measured on unseen data. The evaluation considered instance-level detection, accounting for the total number of detected caries cases, and the model's ability to differentiate between different lesion types. The assessment was conducted across multiple categories, reflecting the model's ability to handle variations in lesion appearance and severity.

## 3. Results

Reliability tests based on spatial overlap of the segmented lesion boundaries yielded an interrater average IoU of 0.82 ± 0.05 and a mean DSC of 0.85 ± 0.04, indicating a high degree of agreement between annotators. The intrarater reliability assessment showed a mean IoU of 0.84 and DSC of 0.87, further confirming the consistency and reproducibility of the annotations.

The final dataset consisted of 730 bitewing radiographs (493 molars and 237 premolars), including 1115 carious lesions categorized as follows: RA1 and RA2 (*N* = 36,933% of the sample), RA3 (*N* = 28,525.5%), RB4 and RC5 (*N* = 27,224.5%), and RC6 (*N* = 18,917%). These images underwent preprocessing and annotation before being used for model training.

After applying image augmentation techniques to enhance variability and improve model generalization, the dataset expanded to a total of 2190 images, which included 3345 carious lesions. To ensure the reliability of the manual annotations used for model training, interrater agreement was assessed using a subset of 10 bitewing radiographs independently annotated by both dentists.

The YOLO11s-seg model was trained for 50 epochs using a batch size of 16 and an image input size of 320 × 320 pixels. The training was conducted on a Tesla T4 GPU with AMP enabled, optimizing memory usage and processing speed.

Throughout training, key loss metrics including box loss, segmentation loss, classification loss, and distribution focal loss, which steadily decreased, indicating effective learning. The overall performance of the model on the training dataset was found to have a P of 66.9, R of 66.2, and mAP50 of 66.8. The final validation phase was conducted on 147 unseen images, assessing the model's detection and segmentation performance across different caries types. The evaluation metrics includedP, R, and mAP50 for segmentation masks, as shown in Table 1. Estimated standard deviations (±SD) for each class's mAP50 reflect model variability over training epochs. These were approximated from terminal logs and training dynamics, indicating stable and consistent model performance.

According to Table 1, the final model demonstrated strong performance, particularly for the RB4 + RC5 and RC6 classes, achieving mAP50 values of 0.74 and 0.80, respectively. This indicates high detection accuracy for these lesion types. In contrast, the RA1 + RA2 and RA3 classes exhibited moderate performance, with lower P and R values. To address this, further improvements such as additional data augmentation and fine-tuning could enhance segmentation accuracy for these classes. The mask-based segmentation performance followed a similar trend, with RC6 achieving the highest segmentation accuracy (mAP50 = 0.80), while RA3 recorded the lowest (mAP50 = 0.39).

**Figure 1:**
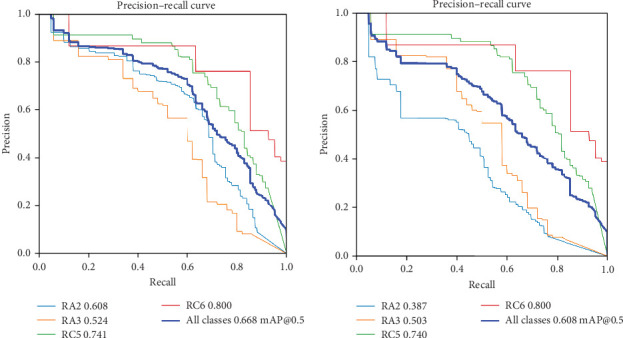
Precision–recall curves for caries detection and segmentation performance. (A) Precision–recall curve for bounding box detection and (B) precision–recall curve for mask-based segmentation.

Additionally, the model's performance was evaluated using precision-recall (PR) curves for both bounding box detection and mask-based segmentation tasks.  Figure 1A,B illustrates the trade-off between P and R across different confidence thresholds, providing a comprehensive understanding of the model's detection and segmentation capabilities.

For bounding box detection, the PR curve (Figure 1A) demonstrates that the model achieves a mAP of 0.668 at an IoU threshold of 0.5 across all classes. P values range from 0.2 to 0.8, while R values span from 0.2 to 0.8, indicating a balanced performance in localizing objects within the images. Specific classes exhibit varying levels of P and R, with some classes showing particularly strong performance. For instance, the high P and R values for classes like RC5 (0.74) and RC6 (0.80) suggest that the model is highly accurate in detecting these specific lesion types. This variability in performance across classes is expected, as some lesion types may inherently present more challenging detection scenarios.

**Figure 2:**
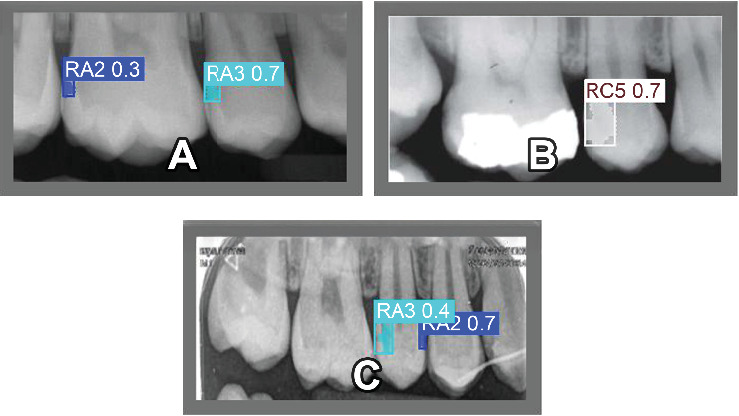
Detection and segmentation results on the test dataset. (A and B) Correctly detected lesions. (C) A misclassification where an RC5 lesion was labeled as RA3.

In the case of mask-based segmentation, the PR curve (Figure 1B) reveals that the model achieves a mAP of 0.608 at an IoU threshold of 0.5 across all classes. P and R values follow a similar trend to the bounding box detection, with P ranging from 0.2 to 0.8 and R from 0.2 to 0.8. While the mAP for segmentation is slightly lower than that of bounding box detection, this is consistent with the increased complexity of pixel-level segmentation tasks compared to object detection.  Figure 2A,B presents representative examples of the model's detection and segmentation performance on the test dataset, primarily illustrating correctly identified carious lesions.  Figure 2C demonstrates a misclassification case, in which an RC5 lesion was erroneously detected and segmented as an RA3 lesion. This example underscores the challenges in differentiating between caries stages with similar radiographic features.

The model still demonstrates strong performance, particularly for certain regions, as evidenced by the higher P and R values for specific classes. For example, the segmentation accuracy for classes like RC5 (0.74) and RC6 (0.80) indicates that the model can achieve high-quality segmentation results in these cases.

## 4. Discussion

This study is among the first to apply YOLOv11 within the standardized ICCMS framework, potentially establishing new avenues for radiographic caries detection following clinical parameters. The model demonstrated high accuracy in detecting advanced dentinal lesions (RC5 and RC6), with mAP50 values of 0.74 and 0.80, respectively, reflecting its strength in identifying pronounced structural changes. In contrast, the detection of early-stage lesions (RA1–RA3) was less successful due to minimal demineralization, subtle radiolucency, and anatomical overlap, factors that inherently limit sensitivity in both clinical and automated diagnosis [27, 28]. Although our model achieved moderate mAP50 scores for these categories (0.52–0.61), these values are in line with or exceed those reported in previous YOLO-based studies [16, 24]. A key distinction in our methodology was the deliberate separation of RA1–RA2 from RA3. While this added complexity may have impacted detection metrics, it enhances clinical applicability by distinguishing lesions typically managed preventively (RA1–RA2) from those that may require restorative treatment (RA3) [13, 29, 30]. This granularity supports more refined diagnostic and treatment decisions, especially for less experienced practitioners.

The difficulty in detecting early-stage lesions (RA1–RA2) is a clinically relevant limitation observed in most caries detection studies. Radiographic evidence alone may not suffice for accurate diagnosis, and AI algorithm alone cannot replace clinical judgement. Several studies have demonstrated that even experienced clinicians exhibit limited diagnostic accuracy for incipient proximal caries on bitewing radiographs, with reported sensitivities as low as 10%–30% and inter-observer agreement often falling below acceptable thresholds [30, 31]. These underscore the inherent challenges of detecting early-stage caries using imaging alone. Therefore, we reinforce the role of AI as a second opinion and a decision-support tool to enhance clinicians' diagnostic performance [32], particularly for subtle lesions, rather than replacing their judgment.

In addition to radiographic limitations, technical factors may have contributed to reduced detection performance on incipient decay. RA3, as a standalone class, had fewer annotated examples, potentially affecting model generalization due to class imbalance. Moreover, annotation of incipient lesions presents inherent challenges (the same as clinically) due to their subtle appearance and lack of clear cavitation on the interproximal enamel, even for experienced clinicians [14, 33]. Finally, YOLO-based architectures, though optimized for real-time detection, may have limited receptive field resolution for detecting small or low-contrast lesions, an architectural constraint noted in prior medical imaging research [24, 34]. Nevertheless, YOLOv11 outperformed earlier YOLO versions and conventional diagnostic methods, reinforcing its potential for real-time clinical applications in dentistry. Segmentation results followed a similar trend, with RC6 yielding the highest accuracy (mAP50 = 0.80), enhancing diagnostic support via precise lesion visualization [26]. These findings suggest that YOLOv11 surpasses previous DL models, particularly in detecting deep dentinal lesions, offering a reliable AI-driven approach for improving accuracy, speed, and efficiency in radiographic caries detection [29, 35].

While this study did not introduce architectural changes to the YOLOv11 model, its selection was driven by recent enhancements that support small-object detection and real-time analysis in noisy imaging environments. Modules such as C3k2, SPFF, and C2PSA improve the model's ability to detect subtle features, an essential requirement for identifying early carious lesions in bitewing radiographs [26, 36]. Moreover, our classification strategy distinguishes RA1–RA2 from RA3, offering a more clinically actionable output aligned with ICCMS recommendations, which is not commonly addressed in earlier models [32, 37]. This decision, though more challenging from a machine learning standpoint, was driven by clinical utility: RA1–RA2 lesions are typically managed preventively, while RA3 lesions may involve cavitation and often require operative treatment [13, 38].

The effectiveness of DL models in caries detection is well-documented, demonstrating notable diagnostic accuracy. However, research has identified limitations with panoramic radiographs for interproximal caries detection, primarily due to image distortions and overlaps inherent to this imaging method. These issues can lead to missed detections, especially for early-stage caries, highlighting a significant challenge for AI applications relying on such radiographic data [39, 40]. Our results are consistent with other YOLO-based studies. For example, Panyarak et al. [24] found YOLOv7 to be more accurate than YOLOv3 for bitewing radiograph analysis. Similarly, YOLOv11's improvements including the C3k2 block for efficient pattern learning and the C2PSA module for focused spatial attention, further enhance detection accuracy while maintaining computational speed [26].

A complementary study by Panyarak et al. [41] evaluated ResNet within the ICCMS RSS, which yielded moderate accuracy but struggled with complex classification tasks. In contrast, YOLOv11′s advanced architecture enabled superior performance across all lesion severities. Nonetheless, early dentinal lesion detection (RA3) remains one of the most challenging diagnostic tasks for both clinicians and AI models. Şenel et al. [30] reported only 17.8% detection accuracy for RA3 lesions by clinicians, compared to 40.2% for deeper lesions (RC5). AI-based approaches, including recent YOLOv8 models, have demonstrated effective interproximal caries detection, though challenges remain for subtle radiolucencies in early lesions like RA3 compared to RB4 and RC5 [42]. Factors contributing to these difficulties include low radiographic contrast and minimal demineralization [30], enamel masking and the absence of clear cavitation [43, 44], and superimposition of adjacent teeth, especially in posterior areas [29, 45]. Urzúa et al. [29] highlighted that overlapping structures significantly reduce RA3 detection accuracy, while variability in clinician interpretation adds further complexity [36, 45].

Although enamel caries (RA1 and RA2) has historically shown low detection rates such as only 10.6% in radiographs according to Şenel et al. [30], our model achieved a substantially higher detection performance (0.608), outperforming many prior reports. Nonetheless, RA3 lesion detection still requires improvement. Studies like those by Urzúa et al. [29] and Serban et al. [35] have emphasized high false-negative rates and limited sensitivity for early enamel lesion detection using conventional radiographic methods. Serban et al.'s [35] systematic review concluded that fluorescence-based methods outperform both radiographic and AI-based models for noncavitated dentin lesions due to poor radiographic contrast.

Overall, the model demonstrated robust performance in both object detection and segmentation tasks. The balanced PR trade-off and high mAP values across most classes support its suitability for clinical use, although typical variability across lesion types remains, especially in early-stage detection. A limitation of this study is the absence of simultaneous clinical assessments, particularly relevant for early-stage lesions (RA1–RA2), where radiographic findings alone may not be sufficient to determine lesion activity or cavitation. However, our classification approach was intentionally based solely on radiographic criteria, in accordance with the ICCMS radiographic scoring framework [13]. This system was developed specifically to enable standardized lesion classification based on bitewing radiographs, especially in contexts where clinical examination is not feasible. Our goal was to train an AI model that replicates radiographic interpretation within this framework. While clinical validation is important for comprehensive diagnosis, it was beyond the scope of this study. Prior studies have shown meaningful correlations between ICCMS radiographic scores and clinical findings, further supporting the clinical relevance of our radiograph-based model [29, 38]. Previous studies have reported that RA1 and RA2 lesions are often noncavitated and may not require restorative treatment [13]. To mitigate diagnostic uncertainty, we adopted a two-step expert annotation protocol, initially by a general dentist and then confirmed by a specialist in caries diagnosis.

Future work could investigate advanced strategies such as focal loss reweighting, synthetic oversampling, or multiscale feature aggregation to enhance early lesion detection performance. Additionally, the current study lacks direct comparison with alternative DL architectures, including U-Net, Faster R-CNN, or transformer-based models which should be incorporated in future work to broaden benchmarking and contextual performance insights. Although YOLOv11 was selected for its state-of-the-art performance and real-time inference capabilities, comparative evaluations with alternative models are essential. Further directions should also include multimodal validation frameworks that integrate clinical and radiographic assessments, as well as analyses of augmented intelligence's impact on improving diagnostic performance among students and clinicians. Emphasis should be placed on evaluating how these AI tools can support and enhance clinician diagnostic accuracy, ultimately advancing real-world decision-making and patient outcomes.

## 5. Conclusion

The YOLOv11 model demonstrated strong performance in detecting advanced carious lesions (RB4 + RC5 and RC6) and fair performance in identifying early-stage lesions (RA1–RA2 and RA3) based on ICCMS radiographic staging. By differentiating noncavitated lesions (RA1–RA2), which typically require preventive care, from potentially cavitated lesions (RA3) that may necessitate restorative intervention, the model provides clinically actionable outputs. Its integration into imaging software, educational platforms, or chairside support tools could assist clinicians, particularly students and early-career practitioners in making more informed treatment decisions and prioritizing early interventions.

## Figures and Tables

**Table 1:** Performance metrics of YOLO-v11 model in detecting various carious lesions.

Class	Precision	Recall	mAP50	mAP ± SD^a^
RA1 and RA2 (RA2)	0.67	0.58	0.61	0.585 ± 0.05
RA3	0.56	0.60	0.52	0.421 ± 0.05
RB4 and RC5 (RC5)	0.81	0.61	0.74	0.784 ± 0.05
RC6	0.64	0.85	0.80	0.803 ± 0.05

^a^SD values are estimated based on variation observed across multiple training epochs and are included to reflect model robustness.

## Data Availability

The data that support the findings of this study are available from the University of Alberta, but restrictions apply to the availability of these data, which were used under license for the current study, and so are not publicly available. Data are, however, available at dentrsch@ualberta.ca upon reasonable request and with permission of the University of Alberta.
